# Deposition of BACE-1 Protein in the Brains of APP/PS1 Double Transgenic Mice

**DOI:** 10.1155/2016/8380618

**Published:** 2016-05-18

**Authors:** Gang Luo, Hongxia Xu, Yinuo Huang, Dapeng Mo, Ligang Song, Baixue Jia, Bo Wang, Zhanqiang Jin, Zhongrong Miao

**Affiliations:** ^1^Department of Neurology, First Affiliated Hospital, Henan University, Kaifeng, Henan 475001, China; ^2^Department of Neurology, Beijing Tiantan Hospital, Capital Medical University, Beijing 100050, China; ^3^Department of Ultrasonic Imaging, Beijing Tiantan Hospital, Capital Medical University, Beijing 100050, China

## Abstract

The main causes of Alzheimer's disease remain elusive. Previous data have implicated the BACE-1 protein as a central player in the pathogenesis of Alzheimer's disease. However, many inhibitors of BACE-1 have failed during preclinical and clinical trials for AD treatment. Therefore, uncovering the exact role of BACE-1 in AD may have significant impact on the future development of therapeutic agents. Three- and six-month-old female APP/PS1 double transgenic mice were used to study abnormal accumulation of BACE-1 protein in brains of mice here. Immunofluorescence, immunohistochemistry, and western blot were performed to measure the distributing pattern and expression level of BACE-1. We found obvious BACE-1 protein accumulation in 3-month-old APP/PS1 mice, which had increased by the time of 6 months. Coimmunostaining results showed BACE-1 surrounded amyloid plaques in brain sections. The abnormal protein expression might not be attributable to the upregulation of BACE-1 protein, as no significant difference of protein expression was observed between wild-type and APP/PS1 mice. With antibodies against BACE-1 and CD31, we found a high immunoreactive density of BACE-1 protein on the outer layer of brain blood vessels. The aberrant distribution of BACE-1 in APP/PS1 mice suggests BACE-1 may be involved in the microvascular abnormality of AD.

## 1. Introduction

Alzheimer's disease (AD) is the major cause of dementia. AD is characterized by progressive memory decline and cognitive impairment [1–3]. The histopathological hallmarks of AD include cerebral amyloid senile plaques, neurofibrillary tangles, and neuronal loss [4–6]. Most cases of AD are sporadic, but about 5–10% of patients have an inherited familial form of the disease (familial Alzheimer's disease, FAD). FAD is caused by the expression of the mutated genes of amyloid precursor protein (APP), presenilin-1 (PS1), and presenilin-2 (PS2) [7]. To date, there is no cure for AD. All of the current medications available treat the symptoms and not the underlying pathology [8]. Therefore, further investigation into the pathogenesis of the disease is needed.

APP is cleaved by sequential actions of *α*-, *β*-, and *γ*-secretases to generate A*β* [9]. While most of the APP protein is processed by *α*-secretases in the nonamyloidogenic pathway, parts of APP are cleaved by *β*-secretase resulting in products of soluble APP-*β* and a C-99 C-terminal fragment. And then the C-99 is further cleaved by *γ*-secretase to produce A*β*-42 [10]. Therefore, *β*- and *γ*-secretase are considered as the enzymes to proteolyze APP and liberate the A*β* peptides [11]. BACE-1, also known as *β*-secretase, is a novel class of type I transmembrane aspartic proteases [11]. BACE-1 exhibits all the functional properties of *β*-secretase and acts as a key enzyme that initiates the formation of A*β*. BACE-1 has a wide distribution in the central nervous system (CNS) with high expression in neurons. After synthesis in the endoplasmic reticulum, BACE-1 is transported to the cell surface where the cleavage of APP occurs [12]. BACE-1 catalyzes the rate-limiting step in A*β* products which are the key players of neurodegenerative cascades in AD [13]. Previous studies demonstrated that suppression of the Wnt pathway resulted in a significant increase of A*β* peptides and expression of BACE-1 [14]. As a secretase, BACE-1 can cleave the membrane-anchored signaling molecule Jagged 1 (Jag1) and BACE-1 null mice that lost the abilities to cleave the Jag1 protein demonstrated enhanced astrogenesis and reduced neurogenesis, which are characteristic features of AD [15]. These findings consistently implied that modifying the activities and protein level of BACE-1 is a possible therapeutic strategy for this devastating neurodegenerative disease. Literatures also showed significant impairment of the neocortical microcirculation in AD transgenic mice and endothelial damage may be an early event of AD pathogenesis [16, 17]. Interestingly, dysfunction of endothelial cells seems to precede plaque deposition in the mouse brain [17]. Furthermore, loss of endothelial NO is also verified to result in increased expression of APP, BACE-1 protein, and A*β* levels in AD mouse brain [17]. Due to the important role of BACE-1 in limiting the generation of beta amyloid, several peptide-based *β*-secretase inhibitors have been described [18]. These inhibitors are developed based on peptide-derived structures, which act as transition state mimetics based on the amino acid sequences at the cleavage site of APP by BACE-1 [19]. However, most of them failed during the preclinical or clinical trials as a treatment for AD so far [19, 20]. Therefore, further deciphering the exact mechanism by which BACE-1 is involved in the pathophysiology of AD is imperative.

The focus of this study was to examine the changes of BACE-1 protein in an APP/PS1 transgenic mouse model. For the first time, we revealed an abnormal distribution of BACE-1 protein in the blood vessels of AD mice, implying an association of BACE-1 protein accumulation with the dysfunction of microvascular units in AD. This study may provide more evidences of how BACE-1 is involved in the earlier stage of AD pathogenesis.

## 2. Materials and Methods

### 2.1. Animals

APP/PS1 double transgenic mice were generated by crossing single transgenic mice expressing human mutant APPK670N/M671N and mutant PS1M146L [21]. After genotyping, 3- and 6-month-old female APP/PS1 mice and age- and gender-matched wild-type (WT) mice were purchased from Zhongke Zasheng Biotech (Beijing, China) and used in the study. The animals were housed at a 12 h day/12 h night light cycle with free access to water and food, under controlled laboratory conditions. All procedures using live animals were approved by the Animal Ethics Committee of Beijing Tiantan Hospital, Capital Medical University, and the Animal Care Committee of Henan University.

### 2.2. Y Maze Test

Spatial working memory was assessed by recording spontaneous alternation behavior during an 8 min session in a Y maze [22]. The sequence of arm entries and total number of entries were recorded. Percentage of alternation was defined as the number of sequential triplets containing entries into all three arms (ABC and ACB constituted a sequential triplet, while A-C-A or A-B-A did not) during the session as a proportion of the maximum possible alternation (equivalent to the total number of arm entries minus −2) × 100. Mice whose total entrance number was less than 15 during the test were not included in data analysis.

### 2.3. Water Maze Test

Reference memory was assessed using the Morris water maze. The procedure was conducted as described in a previous study [22]. In the acquisition (hidden platform) test, the location of the platform was kept constant throughout training. Training consisted of four days of trials. The mouse was released from one of four different quadrants in each trial and was allowed to swim until it climbed onto the platform or reached a maximum time of 60 seconds. Spatial memory was evaluated by assessing the average latency time. A 60-second retention (probe) test was carried out 24 hrs after the last training trial without the platform, during which the time mice spent in the target quadrant (the previous platform location) was recorded.

### 2.4. Immunofluorescence

Animal were perfused with PBS and then fixed with 4% paraformaldehyde. 30 *μ*m brain sections were incubated overnight at 4°C with anti-BACE-1 (1 : 1000, Santa Cruz), CD-31 (1 : 1000, Millipore), or Cystathionine Gamma-Lyase (CG-Lyase) (1 : 1000, Abcam) antibodies, after permeabilization and blocking. The secondary antibodies were appropriate Alexa Fluor 488- or Alexa Fluor 594-conjugated secondary antibodies (Invitrogen-Molecular Probes). Sections were washed, mounted, and examined using an Olympus BX61 fluorescence microscope.

### 2.5. Immunohistochemistry

For immunohistochemistry, four 30 *μ*m coronal brain sections per mice (spaced 180 *μ*m apart) were stained with the ABC Peroxidase Staining Kit (Pierce) in accordance with the manufacturer's instructions. Briefly, endogenous peroxidase activity was blocked by 3% hydrogen peroxide in PBS for 20 min. Sections were blocked at room temperature for 30 min in 5% serum and 0.3% Triton X-100 in PBS and then incubated overnight at 4°C with BACE-1 antibody (1 : 4000, Santa Cruz). Secondary biotinylated antibody was used at a dilution of 1 : 1000 (Vector Laboratories). After being washed 3 times with PBST, the slides were incubated with ABC reagents for 30 min. The chromagen was diaminobenzidine (Thermo Scientific).

### 2.6. Western Blot

Brain tissues were lysed in a cold lysis buffer containing 20 mM Tris-HCl pH 7.6, 150 mM NaCl, 2 mM EDTA, 1 mM EGTA, 0.1% (v/v) Triton X-100, 20 mM NaF, 0.02% NaN3, 1 mM PMSF, and 1% protease inhibitor cocktail (Sigma). The extracted proteins were separated by electrophoresis with the 12% SDS-PAGE and transferred onto nitrocellulose membranes. The target protein of BACE-1 was measured using the primary antibody of anti-BACE-1 antibody (1 : 1000, Santa Cruz) and then the corresponding secondary antibody, followed by development with an ECL kit (PerkinElmer). The antibody against *β*-actin (1 : 4000, Santa Cruz) was used here as an internal control. Quantitative results are expressed as a ratio of BACE-1 to *β*-actin.

### 2.7. Amyloid Plaque

The presence of amyloid plaques in brain sections was investigated after staining with Congo Red kit (Sigma-Aldrich) according to the manufacturer's instructions with minor modification. Briefly, 30 mm sections were stained in Mayer's Hematoxylin Solution for 5 minutes and then rinsed in tap water for 5 minutes. After being incubated in Alkaline Sodium Chloride Solution for 20 minutes, the sections were placed in filtered Alkaline Congo Red solution for another 20 minutes. All sections were rinsed 3 times with absolute ethanol and cleared in xylene before being mounted.

### 2.8. *β*-Secretase Activity

The brain tissue *β*-secretase activity was measured using a fluorometric assay kit purchased from Abcam (ab65357). Briefly, protein was extracted from brain tissue using ice-cold extraction buffer and then centrifuged 10000 ×g for 5 minutes at 4°C. The supernatant was transferred to a new tube and kept on ice. Protein concentration was measured with the BCA kit (Thermo Scientific). A total of 50 *μ*L of sample (total protein 100 *μ*g) was added to each well followed by 50 *μ*L of 2x reaction buffer and 2 *μ*L of *β*-secretase substrate. After incubation in the dark at 37°C for 2 hr, fluorescence was recorded at excitation and emission wavelengths of 355 and 510 nm, respectively, with a Victor*™* X3 Multilabel Plate Reader (PerkinElmer). *β*-secretase activity was expressed as the relative fluorescence units per *μ*g of protein sample.

### 2.9. Statistical Analysis

All data was analyzed using GraphPad PRISM 4.0 software (GraphPad Software). Comparison between groups was performed using ANOVA followed by Newman-Keuls post hoc test or student's *t*-test. Data are shown as mean ± SEM. Differences were considered statistically significant at *P* < 0.05.

## 3. Results

### 3.1. Plaques Deposition and Cognitive Deficiency in APP/PS1 Transgenic Mouse

**Figure 1:**
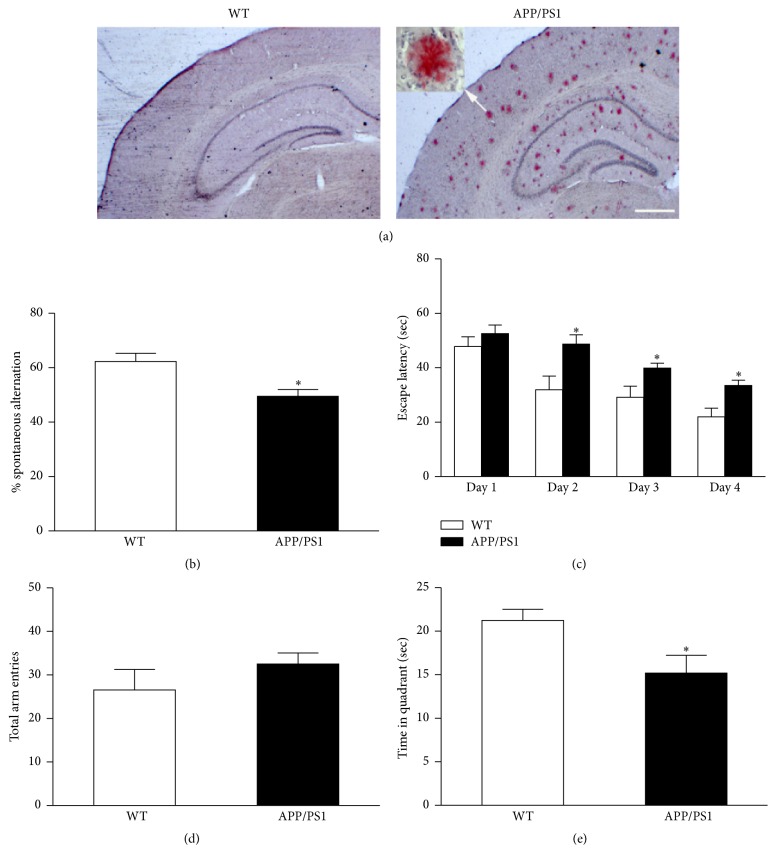
Plaque deposition and memory/cognition deficits in APP/PS1 mice at 6 months of age. Congo Red staining showed the amyloid plaques in the brain of APP/PS1 mice (a). APP/PS1 mice showed reduced spontaneous alternation in Y maze test (b). APP/PS1 mice showed increased trend of total arm entry in Y maze test (c). APP/PS1 mice showed increased escape latency in hidden platform test of water maze compared to WT mice (d). APP/PS1 mice showed less time spent in the target quadrant in probe test of water maze (e). Scale bar represents 500 *μ*m. Data are expressed as means ± SEM. *n* = 7-8, each group. ^*∗*^ *P* < 0.05 versus WT.

AD mice at 6 months of age developed obvious amyloid plaque deposition in the brain as demonstrated with Congo Red staining (Figure 1(a)). This AD-like pathogenesis is consistent with previous report [12]. Spatial working memory was measured with Y maze test. In this test, the ability to alternate requires the mice to remember which arm they have just visited [22]. As shown in Figure 1(b), AD mice demonstrated significantly lower spontaneous alternation compared to WT mice. An increased number of total arm entries were observed in AD mice but did not reach the statistical significance when compared to WT mice (Figure 1(c)).

In the water maze test, spatial memory acquisition was assessed by measuring the escape latency in the hidden platform test [13]. ANOVA analysis showed significantly higher escape latencies for AD mice at days 2, 3, and 4 (Figure 1(d)). Twenty-four hours after the last trial, spatial memory retention was tested by measuring the time spent in the target quadrant where the platform had been located. We found that AD mice spent less time in the target quadrant compared to WT mice (Figure 1(e)).

**Figure 2:**
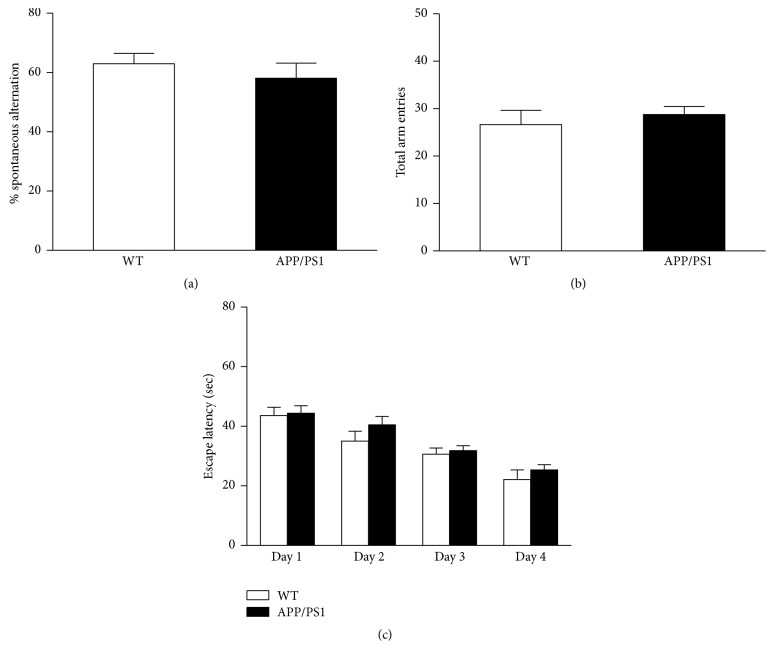
No abnormal behavioral performance was observed in younger APP/PS1 mice (3 months old). There is no significant difference between WT and APP/PS1 mice at 3 months of age on the spontaneous alternation test in Y Maze (a). Total arm entrances showed no significant difference between WT and APP/PS1 mice at 3 months of age in Y maze test (b). Escape latency of hidden platform test in the 3-month-old WT and APP/PS1 mice (c). *n* = 9, each group. Data are expressed as means ± SEM.

The abnormal behavioral performance was not observed in younger APP/PS1 mice (3 months old), although there was a trend towards increased escape times for AD mice during the training stage of the water maze test (Figures 2(a)–2(c)).

### 3.2. BACE-1 Protein Accumulation in the Brain of APP/PS1 Mice

**Figure 3:**
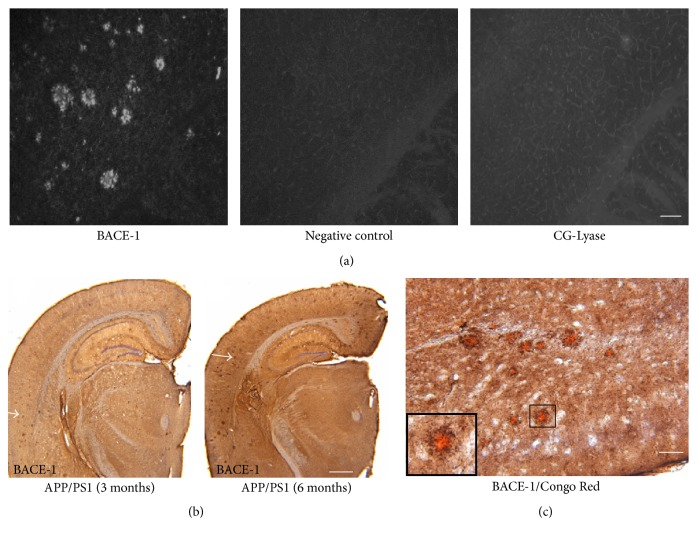
BACE-1 protein accumulating in the brains of APP/PS1 mice. Representative photograph of immunofluorescent staining of BACE-1 protein in the brain cortex of APP/PS1 mice (a). BACE-1 accumulating in the brain of APP/PS1 mice at 3 and 6 months of age (b). Costaining with Congo Red and BACE-1 antibody in the brain cortex of APP/PS1 mice (c). Photograph in the black frame was magnified from the marked areas in the center. Scale bar represents 100 *μ*m (a and c) and 500 *μ*m (b).

*β*-secretase (BACE-1), which performs the rate-limiting step of APP processing during A*β* generation, has become a potential therapeutic target [20]. We examined the BACE-1 protein changes in the brains of AD mice. Using immunofluorescence staining, we found notable protein accumulation of BACE-1 in AD mice (Figure 3(a)). A negative control study was carried out with the same procedure except no primary antibody was used (Figure 3(a)). To further exclude the possibility of nonspecific binding between antibody and the brain section during the staining procedure, CG-Lyase antibody, generated from the same host animals as BACE-1, was used under the same experimental condition as BACE-1 antibody. There was no positive signal detected in brain sections incubated with CG-Lyase antibody (Figure 3(a)). As shown in Figure 3(b), the abnormal BACE-1 accumulation was apparent in brain sections from AD mice at 3 months of age and increased at 6 months of age. These results suggest that the abnormal protein accumulation might be an essential part of the neurodegenerative process of the AD mice. Next, we tested whether there was an association between BACE-1 protein accumulation and amyloid deposition in the mouse brain. Costaining using Congo Red and BACE-1 antibody was used to examine the potential association. We found that all amyloid plaques with Congo Red staining color were surrounded by BACE-1 protein (Figure 3(c)). The results of our above studies showed an aberrant accumulation of BACE-1 protein in the early stages of the disease in this mouse model, which increased rapidly as the mice aged. These results suggest that the accumulated BACE-1 protein might be related to *β* amyloid deposition in the pathogenesis of AD.

### 3.3. BACE-1 Protein Accumulates in the Microvascular Units APP/PS1 Mice Coincidental with the Abnormal Expression of CD31

**Figure 4:**
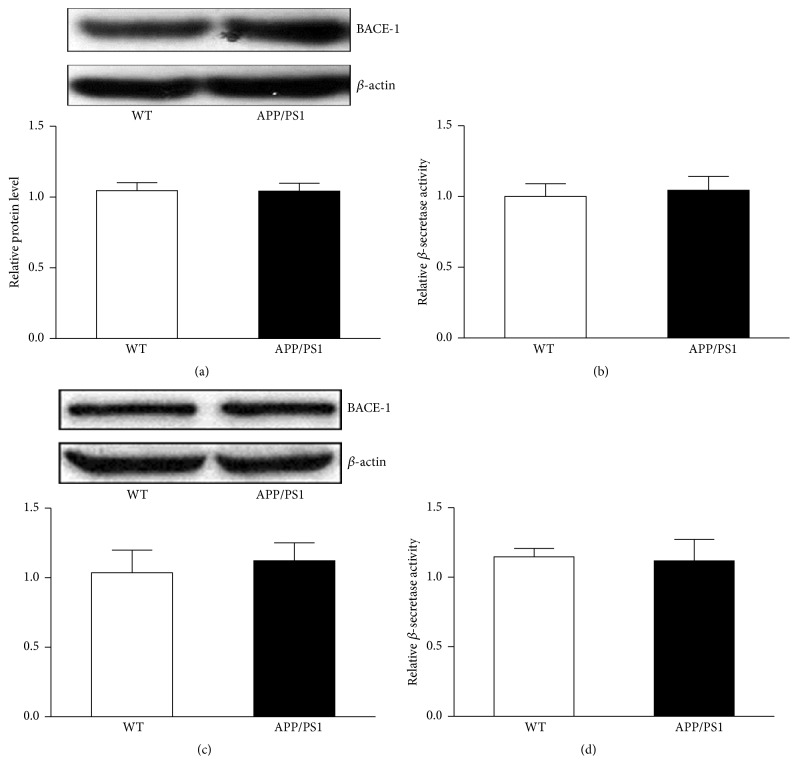
No change on BACE-1 protein expression level was observed in the brains of WT and APP/PS1 mice. Western blot showed comparable expression level of BACE-1 in the brain of WT and APP/PS1 mice at 3 months of age (a). Relative *β*-secretase activity in the brain of WT and APP/PS1 mice was expressed (3 months old) (b). Western blot showed comparable expression level of BACE-1 in the brain of WT and APP/PS1 mice at 6 months of age (c). Relative *β*-secretase activity in the brain of WT and APP/PS1 mice was expressed (6 months old) (d). Data are expressed as means ± SEM. *n* = 5, each group.

**Figure 5:**
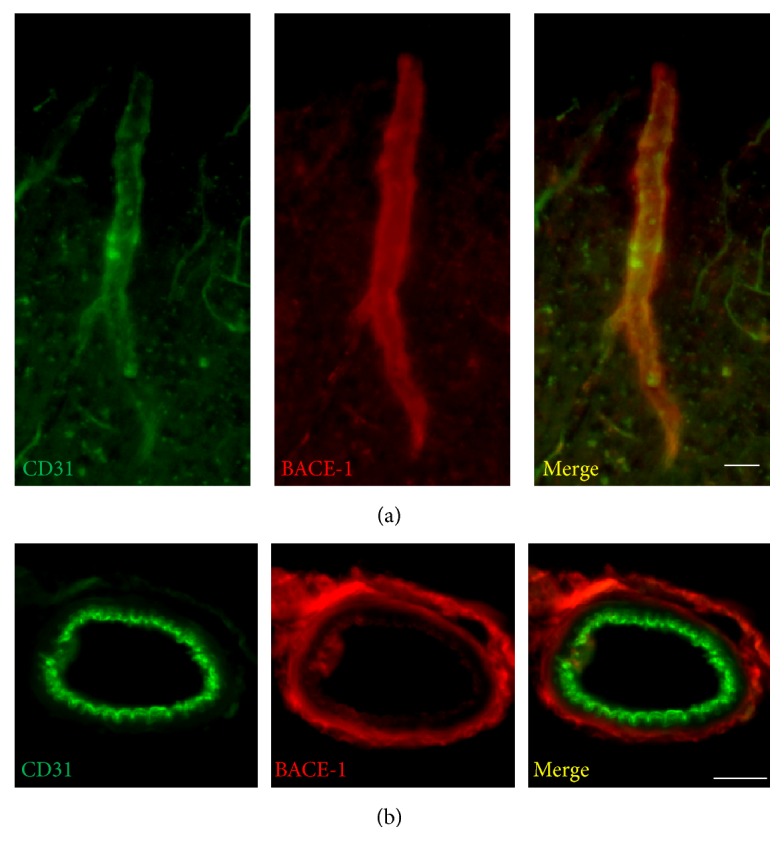
Accumulation of BACE-1 protein in the brain blood vessel in the cortical area of APP/PS1 mice at 3 months of age. High intensity of BACE-1 staining in the blood vessel (a). BACE-1 accumulating in the outer layer of blood vessel of the cortical area of APP/PS1 mice at 3 months of age (b). Scale bar represents 20 *μ*m (a) and 5 *μ*m (b).

The abnormal expression of BACE-1 protein observed in AD mice led us to investigate whether the protein accumulation in these areas was attributed to upregulation of protein expression. Western blot analysis was performed using an antibody specific to BACE-1. No significant differences in protein expression levels of BACE-1 were found between WT and APP/PS1 mice at 3 and 6 months of age (Figures 4(a) and 4(c)). With a fluorometric assay kit, the corresponding *β*-secretase activity was measured in WT and APP/PS1 mice at 3 and 6 months of age. The results showed comparable *β*-secretase activity level between WT and APP/PS1 mice at 3 and 6 months of age (Figures 4(b) and 4(d)). Immunofluorescence staining was further carried out on brain sections to examine the distribution of BACE-1 protein in AD mouse brain. CD31 antibody was used to label the blood vessels. We found high immunoreactivity of BACE-1 protein along the blood vessels (Figure 5(a)). On transverse sections of the vessels, coimmunofluorescence staining revealed notable accumulation of BACE-1 on the outer layer (Figure 5(b)). In summary, our results showed an obvious increase of the immunoreactivity of BACE-1 in the outer layer of blood vessels with immunofluorescence staining. These results may not be due to an increased expression of BACE-1 protein level since there was no significant change in protein levels between WT and AD mice as determined using western blot analysis.

## 4. Discussion

In the amyloidogenic pathway, APP is sequentially cleaved to generate beta peptides. *β*-secretase (BACE-1) works together with other enzymes to cleave APP into A*β* and acts as a rate-limiting enzyme [9]. Also, previous studies have found a dynamic trafficking of BACE-1 between several subcellular locations before it is anchored onto the cell membrane and implies that translocation is the key step for its functional maturity [23].

In the present study, we conducted a detailed examination of BACE-1 expression in an APP/PS1 mouse model. The AD mice developed significant memory/cognitive deficits at 6 months of age with obvious amyloid plaque deposition in the brain. In the same mouse model, aberrant BACE-1 accumulation was observed in brain sections from AD mice at 3 and 6 months of age. The results suggest that BACE-1 protein dysfunction may be involved in the initial stage of the disease. Moreover, our results demonstrated that the increase of protein in the brain might not be linked to the upregulation of protein expression (Figure 4).

Vascular changes are frequently observed in AD brains [24]. Autopsy study demonstrates that the brains of most AD patients exhibit not only the presence of senile plaques and neurofibrillary tangles (NFT), but also the presence of cerebrovascular disease (CVD) [25]. Clinical evidence shows that microvascular degeneration worsens symptoms of dementia in AD patients [26]. In addition, AD and CVD have been found to share many risk factors [27]. Research has shown that microvascular dysfunction contributes to the pathogenesis of AD and may precede neuronal damage and dementia. A recent report validated that hypoxic condition activated cellular responses mainly controlled by hypoxia-inducible transcription factor-1 (HIF-1). Particularly, the oxygen responsive HIF-1*α* subunit may significantly contribute to the cognitive decline by influencing some mechanisms associated with APP amyloidogenic metabolism [28]. But the exact underlying mechanism by which cerebrovascular pathology is initiated is unclear. Some studies report that defective clearance of amyloid may lead to amyloid angiopathy that results in hypoperfusion of brain, which in turn adversely affects formation and absorption of CSF thereby altering clearance of amyloid and promoting vascular and parenchymal deposition [29]. Deposition of amyloid in the walls of medium- and small-size brain vessel is the key feature of cerebral amyloid angiopathy (CAA) [30].

Here, we report a possible new mechanism of angiopathy by focusing on the abnormal distribution of BACE-1 in the earlier stage of AD mouse brains. The present study suggested that BACE-1 deposition might also play an important role by deteriorating the microvascular unit in the initial stage of AD.

Our results are not consistent with previous reports that indicate that BACE-1 protein is elevated in the brain in the late, not early stage of AD [31, 32]. However, in those studies, the focus was on the protein level of BACE-1 without measuring the protein distribution. In the CNS, BACE-1 undergoes intracellular transportation in the cells after synthesis via the secretory pathway [33, 34]. These results imply that translocation may be an essential part of BACE-1 function.

So far, despite promising advances in the understanding of the pathophysiology during the past decades, there are no medical approaches that can cure AD yet. All current medications available for the treatment of the disease only produce limited clinical benefits. As a result, compounds that interfere with proteases that regulate the A*β* products, including the inhibitors of *β*-secretase, were vigorously developed [35]. Consistently, several studies have shown that silencing BACE-1 can reduce A*β*-42 production and therefore blocking this secretase represents a promising therapeutic strategy for AD [10]. However, most of compounds of blocking *β*-secretase fail in preclinical or clinical studies and only one compound (CTS21166) is under ongoing clinical investigation now [35]. Although many of these BACE-1 inhibitors have dropped out, it is still too early to conclude that BACE-1 inhibitors will not be promising tools for the prevention or treatment of AD. The possible explanation for those frustrating results is the inappropriate intervention time courses reemployed in those preclinical or clinical studies since the time point of abnormal activation of BACE-1 in AD is not clearly understood now. To our knowledge, we are the first to show an abnormal distribution of BACE-1 protein in the blood vessels of AD mice, implying an association between BACE-1 protein accumulation and microvascular dysfunction in the early stage of AD. More importantly, our results support the idea that abnormal BACE-1 expression in the microvascular vessel precedes plaque deposition. So this protein deposition in the vascular system may initiate the *β* amyloid cascade in the AD brain. It is hoped that a clinical therapeutic window can be achieved by further investigating the appropriate intervention time in animal model for BACE-1 based therapeutic strategy. Meanwhile, future strategies for developing new drugs should focus on the mechanism of how the aberrant distribution occurs and what significant effects it may exert on the microvascular units.

## 5. Conclusion

Collectively, the present study suggests that BACE-1 protein accumulates in the blood vessels of APP/PS1 mouse brain and indicated the possible involvement of BACE-1 protein on triggering the AD-like pathological changes in this AD mouse model.

## Abbreviation

AD: Alzheimer's disease
FAD: Familial Alzheimer's disease
BACE-1: Beta-site amyloid precursor protein cleaving enzyme-1
APP: Amyloid precursor protein
PS1: Presenilin-1
CD31: Cluster of differentiation 31
CNS: Central nervous system
WT: Wild-type.

## References

[1] Cantillon M., De La Puente A. M., Palmer B. W. (1998). Psychosis in Alzheimer's disease. *Seminars in Clinical Neuropsychiatry*.

[2] Scharre D. W., Chang S.-I. (2002). Cognitive and behavioral effects of quetiapine in Alzheimer disease patients. *Alzheimer Disease and Associated Disorders*.

[3] Kawas C. H., Corrada M. M. (2006). Alzheimer's and dementia in the oldest-old: a century of challenges. *Current Alzheimer Research*.

[4] Kaushal A., Wani W. Y., Anand R., Gill K. D. (2013). Spontaneous and induced nontransgenic animal models of AD: modeling AD using combinatorial approach. *American Journal of Alzheimer's Disease and Other Dementias*.

[5] Hoshi A., Yamamoto T., Shimizu K. (2012). Characteristics of aquaporin expression surrounding senile plaques and cerebral amyloid angiopathy in Alzheimer disease. *Journal of Neuropathology and Experimental Neurology*.

[6] Suh Y.-H., Checler F. (2002). Amyloid precursor protein, presenilins, and *α*-synuclein: molecular pathogenesis and pharmacological applications in Alzheimer's disease. *Pharmacological Reviews*.

[7] Wu L., Rosa-Neto P., Hsiung G.-Y. R. (2012). Early-onset familial alzheimer's disease (EOFAD). *Canadian Journal of Neurological Sciences*.

[8] Iqbal K., Liu F., Gong C.-X. (2014). Alzheimer disease therapeutics: focus on the disease and not just plaques and tangles. *Biochemical Pharmacology*.

[9] O'Brien R. J., Wong P. C. (2011). Amyloid precursor protein processing and Alzheimer's disease. *Annual Review of Neuroscience*.

[10] Jain P., Wadhwa P. K., Rohilla S., Jadhav H. R. (2016). Rational design, synthesis and in vitro evaluation of allylidene hydrazinecarboximidamide derivatives as BACE-1 inhibitors. *Bioorganic & Medicinal Chemistry Letters*.

[11] Venugopal C., Demos C. M., Jagannatha Rao K. S., Pappolla M. A., Sambamurti K. (2008). Beta-secretase: structure, function, and evolution. *CNS & Neurological Disorders—Drug Targets*.

[12] Capell A., Steiner H., Willem M. (2000). Maturation and pro-peptide cleavage of *β*-secretase. *The Journal of Biological Chemistry*.

[13] Marwarha G., Raza S., Meiers C., Ghribi O. (2014). Leptin attenuates BACE1 expression and amyloid-*β* genesis via the activation of SIRT1 signaling pathway. *Biochimica et Biophysica Acta (BBA)—Molecular Basis of Disease*.

[14] Parr C., Mirzaei N., Christian M., Sastre M. (2015). Activation of the Wnt/*β*-catenin pathway represses the transcription of the *β*-amyloid precursor protein cleaving enzyme (BACE1) via binding of T-cell factor-4 to BACE1 promoter. *The FASEB Journal*.

[15] He W., Hu J., Xia Y., Yan R. (2014). *β*-Site amyloid precursor protein cleaving enzyme 1(BACE1) regulates Notch signaling by controlling the cleavage of Jagged 1 (Jag1) and Jagged 2 (Jag2) proteins. *The Journal of Biological Chemistry*.

[16] Iadecola C., Zhang F., Niwa K. (1999). SOD1 rescues cerebral endothelial dysfunction in mice overexpressing amyloid precursor protein. *Nature Neuroscience*.

[17] Austin S. A., Santhanam A. V., Hinton D. J., Choi D.-S., Katusic Z. S. (2013). Endothelial nitric oxide deficiency promotes Alzheimer's disease pathology. *Journal of Neurochemistry*.

[18] Vassar R. (2014). BACE1 inhibitor drugs in clinical trials for Alzheimer's disease. *Alzheimer's Research and Therapy*.

[19] Panza F., Solfrizzi V., Frisardi V. (2009). Disease-modifying approach to the treatment of alzheimers disease: from *α*-secretase activators to *γ*-secretase inhibitors and modulators. *Drugs and Aging*.

[20] Ghosh A. K., Brindisi M., Tang J. (2012). Developing *β*-secretase inhibitors for treatment of Alzheimer's disease. *Journal of Neurochemistry*.

[21] Wong T. P., Debeir T., Duff K., Cuello A. C. (1999). Reorganization of cholinergic terminals in the cerebral cortex and hippocampus in transgenic mice carrying mutated presenilin-1 and amyloid precursor protein transgenes. *The Journal of Neuroscience*.

[22] Kitanaka J., Kitanaka N., Scott Hall F. (2015). Memory impairment and reduced exploratory behavior in mice after administration of systemic morphine. *Journal of Experimental Neuroscience*.

[23] Das U., Scott D. A., Ganguly A., Koo E. H., Tang Y., Roy S. (2013). Activity-induced convergence of APP and BACE-1 in acidic microdomains via an endocytosis-dependent pathway. *Neuron*.

[24] Borroni B., Akkawi N., Martini G. (2002). Microvascular damage and platelet abnormalities in early Alzheimer's disease. *Journal of the Neurological Sciences*.

[25] Honjo K., Black S. E., Verhoeff N. P. L. G. (2012). Alzheimer's disease, cerebrovascular disease, and the *β*-amyloid cascade. *Canadian Journal of Neurological Sciences*.

[26] Snowdon D. A., Greiner L. H., Mortimer J. A., Riley K. P., Greiner P. A., Markesbery W. R. (1997). Brain infarction and the clinical expression of Alzheimer disease: The Nun Study. *The Journal of the American Medical Association*.

[27] Gorelick P. B. (2004). Risk factors for vascular dementia and Alzheimer disease. *Stroke*.

[28] Lonati E., Brambilla A., Milani C., Masserini M., Palestini P., Bulbarelli A. (2014). Pin1, a new player in the fate of HIF-1*α* degradation: an hypothetical mechanism inside vascular damage as Alzheimer's disease risk factor. *Frontiers in Cellular Neuroscience*.

[29] Standridge J. B. (2006). Vicious cycles within the neuropathophysiologic mechanisms of Alzheimer's disease. *Current Alzheimer Research*.

[30] Ghiso J., Frangione B. (2016). Cerebral amyloidosis, amyloid angiopathy, and their relationship to stroke and dementia. *Journal of Alzheimer's Disease*.

[31] Li R., Lindholm K., Yang L.-B. (2004). Amyloid *β* peptide load is correlated with increased *β*-secretase activity in sporadic Alzheimer's disease patients. *Proceedings of the National Academy of Sciences of the United States of America*.

[32] Ahmed R. R., Holler C. J., Webb R. L., Li F., Beckett T. L., Murphy M. P. (2010). BACE1 and BACE2 enzymatic activities in Alzheimer's disease. *Journal of Neurochemistry*.

[33] Brunholz S., Sisodia S., Lorenzo A., Deyts C., Kins S., Morfini G. (2012). Axonal transport of APP and the spatial regulation of APP cleavage and function in neuronal cells. *Experimental Brain Research*.

[34] Rajendran L., Annaert W. (2012). Membrane trafficking pathways in alzheimer's disease. *Traffic*.

[35] Adlard P. A., James S. A., Bush A. I., Masters C. L. (2009). beta-amyloid as a molecular therapeutic target in Alzheimer's disease. *Drugs of Today*.

